# Supplementary material for: Modelling Skylarks (Alauda arvensis) to Predict Impacts of Changes in Land Management and Policy: Development and Testing of an Agent-Based Model
Source: PLoS One. 2013 Jun 6;8(6):e65803. doi: 10.1371/journal.pone.0065803 (PMC3675089; doi:10.1371/journal.pone.0065803)
Supplement: Supporting Information S4 — The skylark ODdox as a zipped archive. (ZIP) [file pone.0065803.s004.zip › Skylark_ODdox/class_t_a_l_ma_s_s_object-members.html]

ALMaSS Skylark ODdox: Member List


ALMaSS Skylark ODdox
 2.0


- Main Page
- Related Pages
- Classes
- Files

- Class List
- Class Index
- Class Hierarchy
- Class Members

TALMaSSObject Member List

This is the complete list of members for TALMaSSObject, including all inherited members.

|  |  |  |
| --- | --- | --- |
| BeginStep(void) | TALMaSSObject | inlinevirtual |
| CurrentStateNo | TALMaSSObject |  |
| EndStep(void) | TALMaSSObject | inlinevirtual |
| OnArrayBoundsError() | TALMaSSObject |  |
| Step(void) | TALMaSSObject | inlinevirtual |
| StepDone | TALMaSSObject |  |
| TALMaSSObject() | TALMaSSObject |  |
| ~TALMaSSObject() | TALMaSSObject | virtual |


- Generated on Thu Jan 10 2013 13:15:38 for ALMaSS Skylark ODdox by
   1.8.1.1
